# Clinical and Radiological Evolution of Bronchiectasis Treated with Long-Term High Flow Nasal Therapy: The Impact of HFT on the Progression of Bronchiectasis

**DOI:** 10.3390/medicina61101807

**Published:** 2025-10-09

**Authors:** Giuseppe Fiorentino, Anna Annunziata, Rosa Cauteruccio, Antonella Marotta, Pasquale Imitazione, Antonietta Coppola, Gerardo Langella, Salvatore Guarino, Francesca Simioli

**Affiliations:** 1 Department of Respiratory Pathophysiology and Rehabilitation, Monaldi Hospital, A.O.R.N. dei Colli, 80131 Naples, Italy; giuseppefiorentino1@gmail.com (G.F.);; 2 Department of Radiology, Monaldi Hospital, A.O.R.N. dei Colli, 80131 Naples, Italy

**Keywords:** High Flow Nasal Cannula, high flow nasal oxygen, HFNO, long-term treatment, chronic obstructive pulmonary disease, asthma, *Pseudomonas aeruginosa*, Bhalla score, mucoid impaction, atelectasis

## Abstract

*Background and Objectives:* a “vicious vortex” model was proposed to explain the pathophysiology of bronchiectasis, incorporating abnormal mucus, altered mucociliary clearance and chronic inflammation. Evidently, airway clearance needs to be implemented in the patient’s daily routine for a protracted period in order to ameliorate the clinical outcomes. High Flow therapy (HFT) has several physiologic effects and represents a valid therapy for various respiratory diseases. The aim of this study is to assess clinical and radiologic effects of long-term HFT in adult non-CF bronchiectasis. *Materials and Methods:* This is a retrospective observational cohort study including adult patients affected by bronchiectasis and frequent exacerbations and hospitalizations. A chest HRCT was performed, and a quantitative evaluation of the scans was conducted applying a modified Bhalla score of five items. A total of 44 patients completed the follow up, 23 in the HF-group and 21 in the controls (No-HF group). *Results:* The median follow up was 41 months (range 36–48 months). The mean age was 65 years, 45% were females. After treatment the annual rate of exacerbations was significantly lower in the HF group (1.2 ± 0.95 versus 3.5 ± 1.0 per year, *p* < 0.0001). The annual rate of hospitalizations was significantly lower in the HF group (0.4 ± 0.52 versus 1 ± 0.93 per year, *p* = 0.01). The total score of the modified Bhalla improved after treatment in the HF group with a mean score of 5.32 versus 8.38, *p* = 0.034. The difference was substantially due to the lower score of mucoid impactions in the HF group. *Conclusions:* Bronchiectasis is an evolutive disease. Long-term HFT reduces the annual rate of exacerbation and hospitalization. In addition, HFT prevents mucoid impaction and potentially influences the radiological evolution of the disease.

## 1. Introduction

Bronchiectasis is a chronic lung disease characterized by abnormal irreversible dilation of bronchi and excessive airway inflammation. This condition is caused by several genetic and acquired medical diseases that lead to substantially the same clinical syndrome. Bronchiectasis overall prevalence in the Italian population referring to General Practitioners (GP) is 163 per 100,000 population, whereas annual incidence is 16.3 per 100,000 person-years [1]. Clinic presentation may vary from chronic cough with augmented sputum production to recurrent respiratory infections. It has a relevant healthcare and social impact, even if the epidemiological burden of the disease is partially unknown with only cross-sectional and retrospective studies been published until now [2]. Moreover, the relevance is often underrated because of coexisting diseases, such as asthma and chronic obstructive pulmonary diseases (COPD).

Lately, a “vicious vortex” model was proposed to explain the pathophysiology of bronchiectasis, incorporating complex interactions among the three components of bronchiectasis: abnormal mucus, altered mucociliary clearance and chronic inflammation [3]. Bronchiectasis is an inflammatory disease and is associated with an imbalance between pro- and anti-inflammatory signaling, leading to the recruitment of inflammatory cells and, ultimately, a self-perpetuating cycle of inflammation [4]. Starting from this point, it is possible to identify successful therapies among mucus clearance techniques, the reduction in inflammation and prevention and control of the infection [5]. Despite this, we can assume that bronchiectasis is a progressive disease, since the natural history of this syndrome is characterized by acute exacerbations, lung damage, airflow limitation, impaired exercise capacity and reduced quality of life.

A fundamental part of the therapeutic approach is airway clearance. Consequently, international guidelines recommend that all patients with bronchiectasis receive instruction in airway clearance techniques taught by respiratory physiotherapists [6]. Unfortunately, evidence in this case is even more fragmentary. Trials of airway clearance techniques are challenging to perform, and compliance is often an insurmountable limit; most studies are below the year. A recent RCT provided more long-term evidence for airway clearance. Munoz et al. [7] compared ELTGOL (slow expiration with the glottis opened in a lateral posture) versus placebo in 22 patients for 12 months. ELTGOL produced higher sputum volume, improvement of quality of life and exacerbations. Notably, the results of the study emphasize that airway clearance needs to be implemented in the patient’s daily routine for a protracted period in order to ameliorate the clinical outcomes.

High Flow Nasal Cannula (HFNC) is a device that delivers heated and humidified air, with a stable fraction of inspired oxygen (FiO_2_) in a range of flow rates up to 60 L per minute (L/m). Therapy with HFNC (HFT) has several physiologic effects, such as humidification and improved mucociliary clearance, washout of dead space, and alveolar recruitment, representing a valid therapy for various respiratory diseases. The importance of mucociliary clearance as a first-line defense mechanism of the bronchial tree is well established. Heated and humidified air possibly enhances the ciliary movement and is a promising approach in patients affected by bronchiectasis non-related to Cystic fibrosis (CF). In a recent crossover study 78 bronchiectasis patients performed HFT for 2 years with a reduction in exacerbations rate and mMRC score. Prominently, the improvement was more consistent in those who tolerated the temperature of 37 °C, independently from the delivered flow [8]. Calabrese et al. reported a significant reduction in exacerbations in 86 non-CF bronchiectasis treated with long-term (LT) HFNC; moreover, the *P. aeruginosa* colonization decreased from 34.9% to 27.9% [9].

## 2. Aim of the Study

The literature lacks evidence about the natural history of bronchiectasis in the very long term, and also the radiologic assessment is not included in recent trials conducted on pharmacological and non-pharmacological therapies. In fact, most studies consider clinical outcomes and bacterial load. The aim of this study is to assess clinical and radiologic effects of LT-HFT in adult non-CF bronchiectasis.

## 3. Materials and Methods

This is a retrospective observational cohort study including patients affected by bronchiectasis syndrome and referring to the Respiratory Pathophysiology and Rehabilitation Unit of Monaldi Hospital in Naples, Italy. We evaluated the clinical and radiological evolution of bronchiectasis for 4 years. Eligible patients with bronchiectasis were recruited from October 2018 to July 2021. We included adult patients (≥18 years) with a consistent diagnosis of bronchiectasis and a compatible HRCT of the chest. Inclusion criteria were a history of frequent exacerbations of at least 3 (>2 per year) and/or at least 1 hospitalization for bronchiectasis (≥1 per year), in the last 12 months. The presence of exacerbations and hospitalizations for bronchiectasis identifies a severe clinical syndrome and implies elevated disease burden. Exacerbations are defined as a deterioration of three or more key symptoms for at least 48 h, in addition to a change in specific treatment. The key symptoms are (1) cough, (2) sputum volume and/or consistency, (3) sputum purulence, (4) breathlessness and/or exercise intolerance, (5) fatigue and/or malaise, and (6) haemoptysis. All patients received the standard of treatment, including mucoregulators, mucolytics, in stable doses throughout at least 3 months prior to enrolment; but also, bronchodilators and inhaled corticosteroids (ICS), as needed. Additionally, all subjects were prescribed periodic physiotherapy. Exclusion criteria were respiratory acidosis (pH < 7.35), hypercapnia (pCO_2_ > 45 mmHg), previous prescription of continuous positive airway pressure (CPAP) or non-invasive ventilation (NIV).

We enrolled 56 patients with a clinical history of exacerbations and consistent use of antibiotics and systemic corticosteroids, despite conventional therapy. The etiology of bronchiectasis was heterogeneous as described in Table 1. Common co-morbidities included systemic hypertension (13 patients), coronary artery disease (4 patients), pulmonary hypertension (6 patients), diabetes mellitus (11 patients), gastroesophageal reflux (14 patients), benign prostatic hypertrophy (8 patients). During the protocol all patients performed at least one on-site visit annually. Additional on-site visits were performed according to clinical symptoms of exacerbation. The chest HRCT was performed at baseline and at the end of follow up, in a stable phase. Other diagnostic and radiologic exams were performed during this time based on the single patient’s necessity. A quantitative evaluation was measured by a modified Bhalla score. It is a 5-item score: severity of bronchiectasis (S), peribronchial thickening (PT), extent of bronchiectasis (E), extent of mucus plugs (MP), collapse or consolidation (C). Every item is assigned a value from 0 to 3, where 0 is no significant alteration, and 3 is severe alteration. The total score (TS) is the sum of the 5 items score, and ranges between 0 and 15. Conventionally a total score up to 5 points indicates a mild radiologic disease, between 6 and 10 points a moderate disease, from 11 to 15 a severe disease. At the end of the study the baseline and follow up CT-scan were evaluated by a single radiologist according to Bhalla score. The radiologist was blind to all clinical and therapeutic data.

28 subjects received additional treatment with HFT (HF group). HFT was started during the acute phase at our clinic, and was eventually prescribed for the long-term use at home based on the patient’s availability to use the device daily as chronic therapy for at least 4 h per day. The enrollment occurred after a run-in period of 2–4 weeks, in order to overcome the acute exacerbation and to acquire a baseline reliable CT-scan. The remaining 28 subjects, who did not perform a sustained and constant HFT, were recorded as the control group (No-HF group).

The aim of this study is to describe the natural evolution of bronchiectasis syndrome both clinically and radiologically over time and compare standard treatment with additional HFT. The follow up was set up to 4 years. 44 subjects completed the follow up, 23 with HFNC, and 21 with standard of care. This study was performed in line with the principles of the Declaration of Helsinki. Patients provided an informed written consent. The study was approved by the local ethic committee.

### 3.1. Statistical Analysis

Data are presented as mean ± standard deviation (SD) for normally distributed continuous variables and as median with interquartile range (IQR) for nonparametric continuous variables. Categorical variables are expressed as numbers (n) and percentages (%). One-way ANOVA (ANalysis of VAriance) with post hoc Tukey HSD (Honestly Significant Difference) were used to determine the homogeneity at baseline and to compare treatments. 95% confidence intervals (95% CI) were calculated. All statistical tests were two-tailed, and *p*-values < 0.05 were considered statistically significant. Statistical analyses were performed using GraphPad Prism (GraphPad Software, version 10.0.2, San Diego, CA, USA). A post hoc power analysis with patients that completed follow up (44) indicated a power of 0.91 (alpha error 0.05).

### 3.2. Study Population

44 patients completed the follow up. The median follow up was 41 months (range 36–48 months). The mean age was 65 years, 45% were females. The mean body mass index (BMI) was 21 kg/m^2^. The vast majority of the patients were former smokers (64%), 5 were current smokers (11%). The most common etiology of bronchiectasis was previous respiratory tract infection (30%); 12 patients were affected by chronic obstructive pulmonary disease (COPD) such as chronic bronchitis and emphysema (27%). 3 patients were affected by asthma and allergic broncho-pulmonary aspergillosis (ABPA) (6.8%), and 2 patients developed bronchiectasis after surgery (4.5%). All patients had a history of frequent exacerbations treated with antibiotics; the mean of exacerbations was 5.7 in the year before enrollment. Hospital admission for exacerbations of bronchiectasis was also frequent with a mean of 1.5 in the year before enrollment. General characteristics are summarized in Table 1.

Baseline pharmacological therapy is reported in Table 2.

## 4. Results

A total of 23 patients received add-on HFNC (HF-group) and completed the follow up. Twenty-one patients completed the follow up with the standard therapy. The annual rate of exacerbations was similar at the baseline between the HF group (5.6 per year ±1.90, 95% CI 4.78–6.42), and the No-HF group (5.8 per year ±1.60, 95% CI 5.07–6.52). After treatment a significant reduction in the number of exacerbations was observed in the HF group (1.2 per year ±0.95, 95% CI 0.79–1.61), compared to the No-HF group (3.5 per year ±1.0, 95% CI 3.45–3.95), *p* < 0.0001. Treatment with HFNC showed a reduction of 65% of exacerbations, compared to the control group.

**Figure 1:**
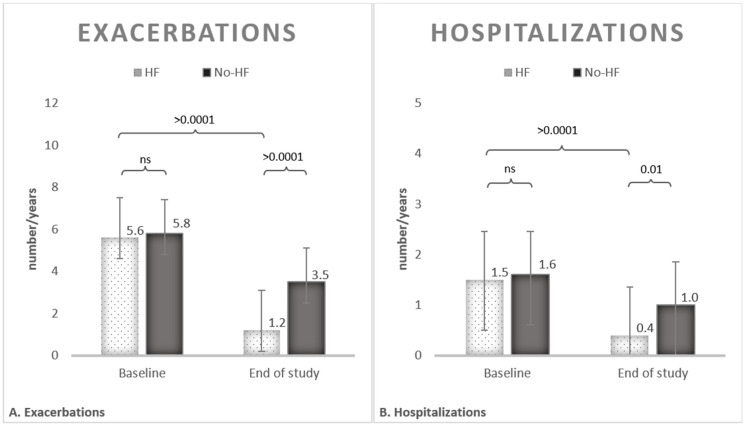
Annual rate of exacerbations (**A**) and hospitalizations (**B**), measured as number of events per year, at baseline (white with dots) and at follow up/end of the study (black).

The annual rate of hospitalizations was comparable at baseline, with a mean in the HF group of 1.5 per year ±0.95 (95% CI 1.09–1.91) and 1.6 per year ±0.85 (95% CI 1.21–1.98) in the No-HF group. After treatment a significant reduction in the number of exacerbations was observed in the HF group, 0.4 per year ±0.52 (95% CI 0.17–0.62), compared to the No-HF group, 1 per year ±0.93 (95% CI 0.57–1.42), *p* = 0.01. Treatment with HFNC showed a reduction of 60% of hospitalizations, compared to the control group. Results are shown in Figure 1.

**Figure 2:**
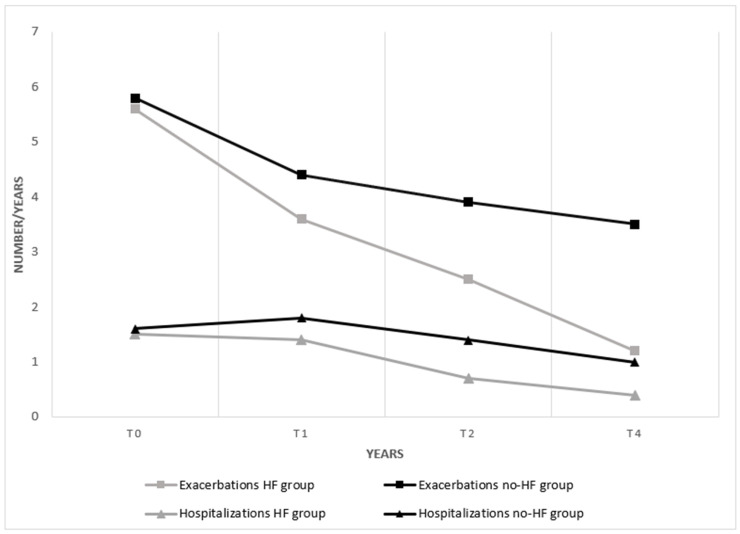
Annual rate of exacerbations (square indicator) and hospitalizations (triangular indicator), measured as number of events per year, during the four years follow up.

The annual rate of exacerbations immediately decreased after HFT and was further reduced after 4 years. The control group also showed a reduction in exacerbations in the first year; successively, the trend was stationary. Similar results were observed on the annual rate of hospitalizations as illustrated in Figure 2.

The HRCT at baseline was evaluated according to a modified Bhalla score. The item-by-item scoring is reported in Table 3. No significant difference was observed at baseline between the HF and No-HF group. No significant difference was observed at baseline in the single items, severity of bronchiectasis (S), peribronchial thickening (PT), extent of bronchiectasis (E), extent of mucus plugs (MP), collapse or consolidation (C). The total score (TS) is equal in the two groups, with a mean of 5.51 ± 3.07. Results of Bhalla are reported in Table 3.

**Figure 3:**
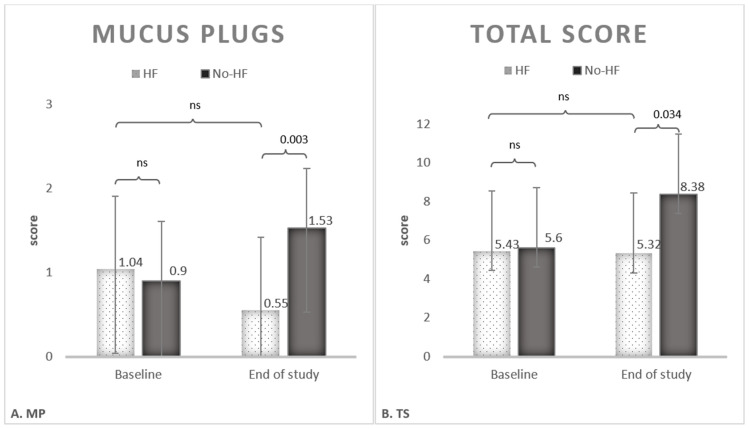
Extent of mucus plugs (**A**) and total score of modified Bhalla (**B**) at baseline (white with dots) and at follow up/end of the study (black).

The radiologic score was applied at the follow up for both groups. The results are listed in Table 4. A significant modification of the modified Bhalla TS was observed after treatment in the HF group with a mean score of 5.32 ± 03.63 versus 8.38 ± 3.91 in the non-treated group, *p* = 0.034. The difference was substantially due to the lower score of MP in the HF group, with 0.551 ± 0.68 versus 1.53 ± 1.17 in the No-HF group, *p* = 0.003. The comparison between the two groups is reported in Table 4. Results on significant Bhalla score are shown in Figure 3.

The TS remained substantially the same after four years in the treated group, while non-treated patients had a progression of 50% compared to baseline. All the other items such as S, MP and C showed a milder, non-significant modification after treatment. On the other hand, we observed an increase in the non-treated group of all items, as follows: S 43%, PT 46%, MP 70%, C 100%.

## 5. Discussion

The results of our study render the evolution of severe bronchiectasis over time. The rate of exacerbations and hospitalization improved in our population; HFT was an effective add-on therapy, with a reduction in exacerbations to below 2 per year, while the control group still presented a clinically significative rate of exacerbations. The result of our study is consistent with a recent systematic review that was conducted on HFNC in the long term (LT-HFNC) (>4 weeks) [10]. This review included chronic respiratory failure and a wide range of chronic respiratory diseases such as COPD, bronchiectasis, ILD, and others; HFNC reduced exacerbations when compared to usual care/home respiratory therapies. Quality of life outcomes were also in favor of HFNC in patients with COPD and bronchiectasis. HFNC had significant effects on hospitalizations, paCO_2_, and lung function. Adherence ranged from 5.2 to 8.6 h/day. In addition, only two of the thirteen reviewed studies exceed the 1 year follow up, and only 3 include bronchiectasis. In fact, international community seems to be skeptical about the topic and bronchiectasis international guidelines do not mention HFNC as a recognized therapy.

Nevertheless, the potential role of HFT has been widely discussed by the Danish respiratory society guideline [11]. According to this document, humidification of the airways is essential to prevent dryness and congestion that cause increase airway resistance and increase the work of breathing; but also, it is important for airway resistance to inflammation and infection, improving the velocity of mucociliary transport. The results of our study support the evidence that HFNC enhances mucociliary clearance and mitigates inflammation, when set at temperature and humidity matching human physiology (37 °C and 100% humidity).

The effectiveness on exacerbations and hospitalizations is obtained with long term use, suggesting that not only HFT helps airways patency, but eventually prevents lung damage progression over time. Indeed, a variable proportion of bronchiectasis is destined to worsen but we have a fragmentary knowledge about how and why this happens. Park et al. reported that age at diagnosis, BMI, chronic infection of *P. aeruginosa* and isolation of nontuberculous mycobacterium in respiratory specimens are related to the Bhalla score change [12]. Our study highlights how the Bhalla total score tends to progress over the four years follow up in the control group despite the standard of care, worsening by 50% compared to baseline; on the other hand, additional HFT avoids the expected worsening of the total score which remains stable in the treated group.

Analyzing the single Bhalla items in the control group, we observed that mucoid impaction increased by 70% and atelectasis/consolidations by 100% compared to baseline; thus, suggesting that mucoid impaction represents the typical evolution of the disease. On the contrary, the extent of bronchiectasis did not differ at the follow up for both HF and Non-HF group, suggesting that clinical progression is not related to subsequent diffusion of bronchiectasis in the lungs. As previously emerged, mucus hypersecretion, abnormal secretions, and decreased clearance can cause obstruction of airways, limit airflow, and accelerate lung function decline, leading to obstructive pulmonary diseases [13]. Even though chronic bronchial diseases share distinct origins and mechanisms, the common pathophysiological and clinical manifestations of these diseases are mucus hypersecretion, airway inflammation, and impaired mucociliary clearance [14]. Interestingly, patients with COPD, CF, non-CF bronchiectasis, and asthma have altered mucins and water content at the airway epithelial surface, higher solid percentage, altered viscosity due to the release of DNA and F-actin filaments from stimulated neutrophils and damaged epithelial cells [15]. It is our belief that the paradigmatic evolution of severe bronchiectasis starts from mucoid impaction to zonal dysventilation, loss of volume and atelectasis. Remarkably, in this study, HFT guaranteed a decrease in mucoid impaction along with reduction in the exacerbation and hospitalization rates.

Secretion management is often a challenge in clinical practice, but it plays a key role in the future approach to severe bronchiectasis. HFNC can be easily implemented in the patient’s daily routine; in can be performed during daytime or nighttime based on preference, but should be further investigated; low to medium flow allows speaking, coughing and sleeping. We recommend the highest temperature and humidity that is tolerated, 37 °C and 100%, respectively.

Likely in our population, the most beneficial results can be observed in more severe patients; besides it is not possible to postulate exactly how many exacerbations can predict efficacy.

### Future Perspectives and Limitations of the Study

Finally, we need more studies on LT-HFT. We can count on limited data in chronic respiratory conditions such as bronchiectasis. Questions need to be answered about the optimal target of population and the correct starting time. Also, a cost–benefit evaluation is missing. The optimal flow is not established; lower flows will probably improve tolerance, and mechanical models suggest better airway clearance at lower flows; however, no studies on higher flows in stable conditions exist [10]. The optimal duration of use has not been investigated in any of the chronic diseases, and it is therefore not known whether it differs between diseases.

This study has several limitations. First of all, the small number of participants is due to the necessity of a control group and the very long follow up. Only very symptomatic patients were motivated to adhere to such a long period of therapy. 12 subjects (21%) were lost to follow up. Theorizing about the natural evolution of a disease requires time, and resources. Moreover, this study was impacted by the global situation between 2020–2021.

Secondly, the lack of a possible placebo is evident. Thirdly, a confounding factor may be represented by the vast heterogeneity of our population, where concomitant diseases such as COPD and asthma play a role in the expected progression of bronchiectasis too. In fact, approximately one third of the study population received bronchodilators according to previous lung function data. But spirometry was not systematically performed during the study and was not included in the outcomes.

Lastly, microbiological analysis was performed only for clinical reasons, and it was not planned per protocol. Infection and colonization by various germs may ineluctably interfere with the evolution of bronchiectasis; besides HFT can itself modify the onset and persistence of infection. From then, this aspect needs to be further investigated in the future.

## 6. Conclusions

Bronchiectasis is an evolutive disease. LT-HFT reduces the annual rate of exacerbation (−65%) and hospitalization (−60%). In addition, HFT prevents mucoid impaction and potentially influences the radiological evolution of the disease.

## Figures and Tables

**Table 1:** General characteristics at baseline of the study population.

	All (n = 44)
**General characteristics**	
Age, years, mean (SD)	65.78 (10.74)
Female, n (%)	20 (45.5)
BMI, kg/m^2^, mean (SD)	21.2 (9.6)
**Smoking history, n (%)**	
Current smokers	5 (11)
Former smokers	28 (64)
Non smokers	11 (25)
**Etiology/underlying disease, n (%)**	
Respiratory infection sequelae	13 (30)
COPD/emphysema	12 (27)
Genetic/congenital	6 (13.6)
Asthma/ABPA	3 (6.8)
Idiopathic	8 (18.1)
Surgery/trauma	2 (4.5)
**Exacerbations, n/per year (SD)**	5.7 (1.8)
**Hospitalizations, n/per year (SD)**	1.5 (0.97)

Legend: BMI: body mass index. COPD: chronic obstructive pulmonary disease. ABPA: allergic broncho-pulmonary aspergillosis.

**Table 2:** Pharmacological therapy at baseline of the study population.

	All (n = 44)	HF (n = 23)	No-HF (n = 21)
LABA, n (%)	14 (32)	8 (35)	6 (29)
LAMA, n (%)	9 (20)	5 (22)	4 (19)
ICS, n (%)	9 (20)	5 (22)	4 (19)
NAC, n (%)	24 (55)	14 (61)	10 (48)
Carbocisteine, n (%)	20 (45)	9 (39)	11 (52)

Legend: LABA: long acting β2 agonist. LAMA: long-acting muscarinic antagonist. ICS: inhaled corticosteroid. NAC: n-acetyl-cysteine.

**Table 3:** Findings with high resolution computed tomography (HRCT) of the chest at baseline, in all patients (All), in the group treated with HFNC (HF) and in the group with conventional therapy (No-HF).

Modified Bhalla Score	All (n = 44)	HF (n = 23)	No-HF (n = 21)	*p*-Value
Severity of bronchiectasis, mean (SD)	1.28 (0.91)	1.17 (0.88)	1.4 (0.94)	0.88
Peribronchial thickening, mean (SD)	1.14 (0.77)	1.13 (0.81)	1.15 (0.74)	0.99
Extent of bronchiectasis, mean (SD)	1.30 (0.94)	1.21 (0.90)	1.4 (0.99)	0.93
Extent of mucus plugs, mean (SD)	0.98 (0.80)	1.04 (0.87)	0.9 (0.71)	0.95
Collapse or consolidation, mean (SD)	0.88 (0.70)	1 (0.74)	0.75 (0.64)	0.75
Total score, mean (SD)	5.51 (3.07)	5.43 (3.11)	5.6 (3.10)	0.99

**Table 4:** Findings with high resolution computed tomography (HRCT) of the chest at follow up, in all patients (All), in the group treated with HFNC (HF) versus in the group with conventional therapy (No-HF).

Modified Bhalla Score	All (n = 44)	HF (n = 23)	No-HF (n = 21)	*p*-Value
Severity of bronchiectasis, mean (SD)	1.63 (1.06)	1.31 (1.00)	2 (1.03)	0.13
Peribronchial thickening, mean (SD)	1.40 (0.98)	1.16 (0.89)	1.68 (1.01)	0.23
Extent of bronchiectasis, mean (SD)	1.51 (0.98)	1.42 (0.96)	1.62 (1.02)	0.91
Extent of mucus plugs, mean (SD)	1.00 (1.05)	0.55 (0.68)	1.53 (1.17)	0.003 **
Collapse or consolidation, mean (SD)	1.20 (0.90)	0.95 (0.97)	1.5 (0.73)	0.13
Total score, mean (SD)	6.71 (4.02)	5.32 (3.63)	8.38 (3.91)	0.034 *

* *p* < 0.05, ** *p* < 0.01.

## Data Availability

The data that support the findings of this study are available on request from the corresponding author, [initials]. The data are not publicly available due to [restrictions, e.g., their containing information that could compromise the privacy of research participants].

## References

[1] Aliberti S., Sotgiu G., Lapi F., Gramegna A., Cricelli C., Blasi F. (2020). Prevalence and incidence of bronchiectasis in Italy. BMC Pulm. Med..

[2] Quint J.K., Millett E.R., Joshi M., Navaratnam V., Thomas S.L., Hurst J.R., Smeeth L., Brown J.S. (2016). Changes in the incidence, prevalence and mortality of bronchiectasis in the UK from 2004 to 2013: A population-based cohort study. Eur. Respir. J..

[3] Flume P.A., Chalmers J.D., Olivier K.N. (2018). Advances in bronchiectasis: Endotyping, genetics, microbiome, and disease heterogeneity. Lancet.

[4] Keir H.R., Chalmers J.D. (2021). Pathophysiology of bronchiectasis. Semin. Respir. Crit. Care Med..

[5] Choi H., McShane P.J., Aliberti S., Chalmers J.D. (2024). Bronchiectasis management in adults: State of the art and future directions. Eur. Respir. J..

[6] Polverino E., Goeminne P.C., McDonnell M.J., Aliberti S., Marshall S.E., Loebinger M.R., Murris M., Cantón R., Torres A., Dimakou K. (2017). European Respiratory Society guidelines for the management of adult bronchiectasis. Eur. Respir. J..

[7] Muñoz G., de Gracia J., Buxó M., Alvarez A., Vendrell M. (2018). Long-term benefits of airway clearance in bronchiectasis: A randomised placebo-controlled trial. Eur. Respir. J..

[8] Simioli F., Fiorentino G., Cauteruccio R., Coppola A., Imitazione P., Marotta A., Di Spirito V., Annunziata A. (2023). Long-Term High Flow Nasal Cannula Therapy in Primary and Secondary Bronchiectasis. Healthcare.

[9] Calabrese C., Nolasco S., Annunziata A., Sola A., Imitazione P., Campisi R., Simioli F., Balestrino M., Ferrentino L., Vancheri C. (2024). Long-Term High-Flow Nasal Therapy in Patients with Bronchiectasis of Different Severity: A Retrospective Cohort Study. J. Clin. Med..

[10] Jácome C., Jácome M., Correia S., Flores I., Farinha P., Duarte M., Winck J.C., Sayas Catalan J., Díaz Lobato S., Luján M. (2024). Effectiveness, Adherence and Safety of Home High Flow Nasal Cannula in Chronic Respiratory Disease and Respiratory Insufficiency: A Systematic Review. Arch. Bronconeumol..

[11] Weinreich U.M., Juhl K.S., Søby Christophersen M., Gundestrup S., Hanifa M.A., Jensen K., Andersen F.D., Hilberg O., Storgaard L.H. (2023). The Danish respiratory society guideline for long-term high flow nasal cannula treatment, with or without supplementary oxygen. Eur. Clin. Respir. J..

[12] Park J., Kim S., Lee Y.J., Park J.S., Cho Y.J., Yoon H.I., Lee K.W., Lee C.T., Lee J.H. (2016). Factors associated with radiologic progression of non-cystic fibrosis bronchiectasis during long-term follow-up. Respirology.

[13] Pangeni R., Meng T., Poudel S., Sharma D., Hutsell H., Ma J., Rubin B.K., Longest W., Hindle M., Xu Q. (2023). Airway mucus in pulmonary diseases: Muco-adhesive and muco-penetrating particles to overcome the airway mucus barriers. Int. J. Pharm..

[14] Fahy J.V., Dickey B.F. (2010). Airway mucus function and dysfunction. N. Engl. J. Med..

[15] Thiam H.R., Wong S.L., Qiu R., Kittisopikul M., Vahabikashi A., Goldman A.E., Goldman R.D., Wagner D.D., Waterman C.M. (2020). NETosis proceeds by cytoskeleton and endomembrane disassembly and PAD4-mediated chromatin decondensation and nuclear envelope rupture. Proc. Natl. Acad. Sci. USA.

